# Is venous congestion associated with reduced cerebral oxygenation and worse neurological outcome after cardiac arrest?

**DOI:** 10.1186/s13054-016-1297-2

**Published:** 2016-05-15

**Authors:** Koen Ameloot, Cornelia Genbrugge, Ingrid Meex, Ward Eertmans, Frank Jans, Cathy De Deyne, Joseph Dens, Wilfried Mullens, Bert Ferdinande, Matthias Dupont

**Affiliations:** Department of Cardiology, Ziekenhuis Oost-Limburg, Schiepse Bos, 3600 Genk, Belgium; Department of Anesthesiology and Critical Care Medicine, Ziekenhuis Oost-Limburg, Genk, Belgium; Faculty of Medicine and Life Sciences, University Hasselt, Diepenbeek, Belgium

**Keywords:** Post-cardiac arrest, Central venous pressure, Cerebral perfusion, Outcome

## Abstract

**Background:**

Post-cardiac arrest (CA) patients are at risk of secondary ischemic damage in the case of suboptimal brain oxygenation during an ICU stay. We hypothesized that elevated central venous pressures (CVP) would impair cerebral perfusion and oxygenation (venous cerebral congestion). The aim of the present study was to investigate the relationship between CVP, cerebral tissue oxygen saturation (SctO_2_) as assessed with near-infrared spectroscopy (NIRS) and outcome in post-CA patients.

**Methods:**

This was an observational study in 48 post-CA patients with continuous CVP and SctO_2_ monitoring during therapeutic hypothermia.

**Results:**

The relationship between CVP and mean SctO_2_ was best described by an S-shaped, third-degree polynomial regression curve (SctO_2_ = −0.002 × CVP^3^ + 0.08 × CVP^2^ – 1.07 × CVP + 69.78 %, *R*^2^ 0.89, n = 1,949,108 data points) with high CVP (>20 mmHg) being associated with cerebral desaturation. Multivariate linear regression revealed CVP to be a more important determinant of SctO_2_ than mean arterial pressure (MAP) without important interaction between both (SctO_2_ = 0.01 × MAP – 0.20 × CVP + 0.001 × MAP × CVP + 65.55 %). CVP and cardiac output were independent determinants of SctO_2_ with some interaction between both (SctO_2_ = 1.86 × CO – 0.09 × CVP – 0.05 × CO × CVP + 60.04 %). Logistic regression revealed that a higher percentage of time with CVP above 5 mmHg was associated with lower chance of survival with a good neurological outcome (cerebral performance category (CPC) 1–2) at 180 days (OR 0.96, 95 % CI 0.92–1.00, *p* = 0.04). In a multivariate model, the negative association between CVP and outcome persisted after correction for hemodynamic variables, including ejection fraction and MAP.

**Conclusions:**

Elevated CVP results in lower brain saturation and is associated with worse outcome in post-CA patients. This pilot study provides support that venous cerebral congestion as indicated by high CVP may be detrimental for post-CA patients.

**Electronic supplementary material:**

The online version of this article (doi:10.1186/s13054-016-1297-2) contains supplementary material, which is available to authorized users.

## Background

After cardiac arrest (CA), patients with suboptimal brain oxygenation during their stay in the intensive care unit have a large cerebral penumbra and risk of secondary ischemic damage [1]. Therefore, thorough knowledge of the physiological determinants of cerebral oxygen supply in the post-CA period is paramount for physicians taking care of these patients. We previously showed that mean arterial pressure (MAP), mixed venous oxygen saturation, cardiac output, hemoglobin, partial pressure of oxygen (PaO_2_), and carbon dioxide (PaCO_2_) are important determinants of cerebral tissue oxygen saturation (SctO_2_) measured with near-infrared spectroscopy (NIRS) [2–4]. Current hemodynamic post-CA guidelines focus on targeting an MAP of 65 mmHg, based on the assumption that this would result in optimal cerebral perfusion assuming normal cerebrovascular autoregulation [5]. However, cerebral perfusion pressure equals MAP minus intracranial pressure (ICP). As it has been shown that there is a good correlation between ICP and central venous pressure (CVP) [6], we hypothesized that there would be negative correlation between CVP and cerebral perfusion (venous cerebral congestion). In this way, cerebral oxygenation might be compromised in after CA, with elevated CVP due to right ventricular failure, fluid overload, mechanical positive pressure ventilation, or pulmonary disease. The aim of the present study was to investigate the relationship between CVP, SctO_2_ as assessed with near-infrared spectroscopy (NIRS), and outcome after CA in patients under therapeutic hypothermia.

## Methods

### Study population

All comatose survivors of non-traumatic CA treated in our tertiary care hospital (Ziekenhuis Oost-Limburg, Genk, Belgium) are prospectively enrolled in our database. Patients could have been resuscitated in hospital, referred by another hospital, or admitted to our own emergency ward. All patients were treated uniformly according to the institutional post-CA protocol. As part of this protocol, patients are routinely monitored by cerebral saturation (SctO2) monitoring on admission to the coronary care unit. For the purpose of the present study, we selected patients with continuous CVP recordings with a 2-sec time interval. Written informed consent was obtained from a next of kin. The local medical ethics committee approved the study protocol (Ziekenhuis Oost-Limburg ethical committee, 15/2/2011).

### General management

Our institutional post-CA protocol has been described previously [2–4]. Briefly, all patients were intubated, mechanically ventilated and sedated with propofol and remifentanil if hemodynamically tolerated. Cisatracurium was administered in the case of problematic ventilation and shivering. Unless an obvious non-cardiac cause could be identified, all patients were referred for urgent coronary angiography followed by percutaneous coronary intervention when indicated. Therapeutic hypothermia was induced shortly after admission by cold saline (4 °C, 30 ml/kg) and further mechanically induced and maintained in the coronary care unit by endovascular (Icy-catheter, CoolGard® 3000, Alsius, Irvine, CA, USA) or surface (ArcticGelTM pads, Arctic Sun® 5000, Medivance, Louisville, CO, USA) cooling systems at 33 °C for 24 h. During their stay in the ICU, hemodynamic management wasa performed according to the guidelines. If signs of inadequate circulation persisted despite correct fluid resuscitation (wedge pressure >18 mmHg), norepinephrine was infused with a target MAP >65 mmHg and subsequently dobutamine to target a cardiac index >2.2 L/min/m^2^. After rewarming (0.3 °C/h) sedation was titrated towards the patient’s comfort with efforts towards minimizing sedation. Patients were extubated when their neurological, respiratory, and hemodynamic status had sufficiently recovered.

### Data collection

Cerebral tissue oxygen saturation was continuously measured with NIRS using the FORE-SIGHT™ technology (CAS Medical systems, Branford, CT, USA). Sensors were bilaterally applied to each frontotemporal area before the start of mechanically induced hypothermia. Sensors were covered to prevent ambient light interference. Cerebral saturation data were not used to guide hemodynamic management. Unless contraindicated or considered inappropriate by treating physicians, all patients were monitored by a new-generation pulmonary artery catheter (CCOmbo PAC®, Edwards Life Science, Irvine, CA, USA) connected to the appropriate monitor (Vigilance II®, Edwards Life Science, Irvine, CA, USA). Central venous pressure and cardiac output were measured continuously by thermodilution. In patients without a pulmonary artery catheter, CVP was measured using the deep vein catheter accessed through the subclavian or jugular vein. MAP was obtained from a radial artery line. Both MAP and CVP were measured at the level of the tragus. Hemodynamic and SctO_2_ data were transmitted electronically to a personal computer with a 2-sec time interval, together with information on blood temperature and oxygen saturation. The ejection fraction was determined by transthoracic echocardiography.

### Statistics

Results are expressed as mean (±SD, standard deviation) unless otherwise stated. All simultaneously obtained CVP-SctO_2_ pairs collected during the first 24 h after ICU admission were pooled. The mean SctO_2_ was calculated per mmHg CVP (range 0–25 mmHg) and per mmHg MAP minus CVP (range 40–90 mmHg). Regression curves were fitted (with calculation of the Pearson correlation coefficient) to describe the relationship between SctO_2_ and CVP. Subsequently, all CVP-SctO_2_ pairs were stratified according to the simultaneously obtained MAP (60–70 mmHg, 70–80 mmHg, 80–90 mmHg, 90–100 mmHg) and cardiac output (2.5–5 L/min, 5–7.5 L/min, >7.5 L/min) and similar regression analysis was performed. Subsequently, two separate multivariate linear regression models were constructed to describe the interactions between MAP and CVP, and cardiac output and CVP, as determinants of SctO_2_. We performed trend analysis by calculating ΔCVP and ΔSctO_2_ during 1-h time intervals during the 24-h study period. Finally, the percentage of time above each CVP value (range 0–25 mmHg) was calculated per patient. A good outcome was defined as survival in cerebral performance category (CPC) 1–2 at 180 days. The CPC score ranges from 1 (good cerebral performance) to 5 (death). Odds ratios (and 95 % CI) were calculated to assess the association between the time above each CVP value and the odds to survive with good neurological outcome. Univariate logistic regression was used to test for significance (assuming that a higher percentage of time spent above an optimal CVP level would negatively affect outcome). Two separate multivariate models were constructed using backward multivariate logistic regression. Cardiac arrest variables (patient age, bystander cardiopulmonary resuscitation (CPR) <10 minutes, shockable rhythm, time to return of spontaneous circulation (ROSC), and percentage time with CVP above 5 mmHg) were included in the first model. Hemodynamic variables (percentage time with MAP <65 mmHg, ejection fraction, and percentage time with CVP >5 mmHg) were included in the second model, Statistical analysis was performed using Matlab software (version R2010b, Mathworks, Natick, MA, USA). A *p* value <0.05 was considered significant.

## Results

### Patients

Forty-eight patients were included in the study. Mean age was 62 ± 13 years, 59 % were male, 84 % received bystander CPR, and 68 % had a shockable rhythm. Cardiac output and filling pressures were continuously monitored with a pulmonary artery catheter in 39 patients and CVP was monitored by a deep vein catheter in the remaining 9 patients. Mean MAP was 77 ± 8 mmHg, mean mixed venous blood oxygen saturation (SVO_2_) 67 ± 10 %, mean cardiac output 3.7 ± 1.2 L/min, and mean CVP 10 ± 4 mmHg. A summary of the baseline characteristics is provided in Additional file 1.

### CVP negatively affects SctO_2_

**Fig. 1:**
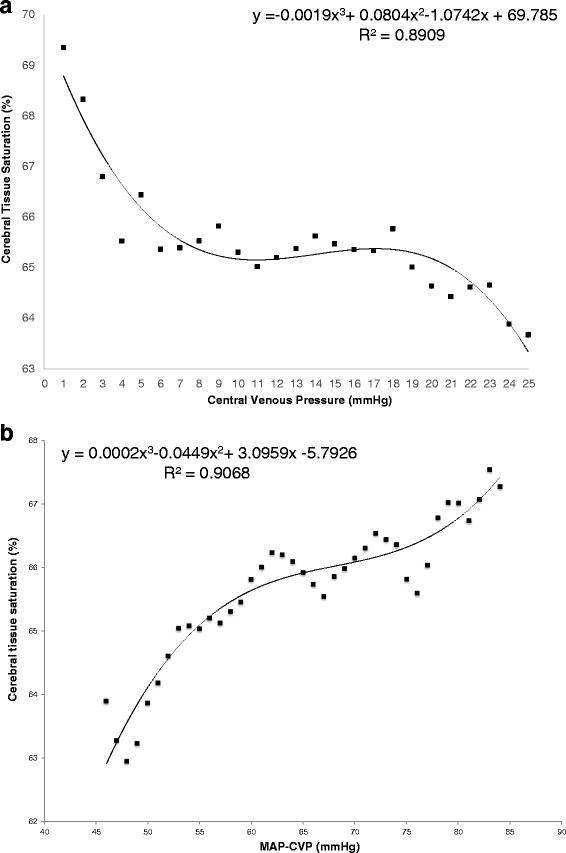
The mean cerebral tissue oxygen saturation (SctO_2_) is shown per mmHg central venous pressure (**a**) and the mean SctO_2_ is shown per mmHg mean arterial pressure-central venous pressure (*MAP-CVP*) (**b**)

The relationship between CVP and mean SctO_2_ was best described by an S-shaped, third-degree polynomial regression curve (SctO_2_ = −0.002 × CVP^3^ + 0.08 × CVP^2^ − 1.07 × CVP + 69.78 %, *R*^2^ 0.89, n = 1,949,108 data points), with low CVP (<5 mmHg) being associated with normal SctO_2_ values and high CVP (>20 mmHg) being associated with cerebral desaturation (Fig. 1a). The relationship between simultaneously obtained MAP minus CVP pairs and mean SctO2 was also best described by an S-shaped, third-degree polynomial regression curve (SctO_2_ = 0.0002 × (MAP-CVP)^3^ –0.0445 × (MAP-CVP)^2^ – 3.0959 × (MAP-CVP) – 5.7926 %, *R*^2^ 0.91) (Fig. 1b).

### CVP is more important than MAP as a determinant of SctO_2_

**Fig. 2:**
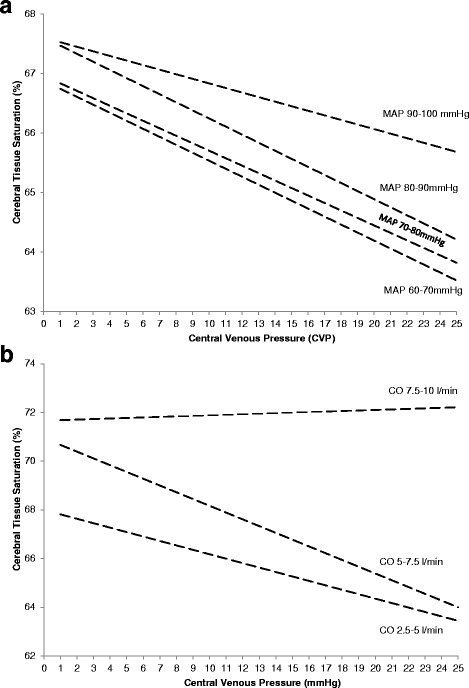
The mean cerebral tissue oxygen saturation (SctO_2_) per mmHg central venous pressure (*CVP*) after stratification of the data points according to the simultaneously obtained mean arterial pressure (*MAP*) (60–70 mmHg, 70–80 mmHg, 80–90 mmHg, 90–100 mmHg) (**a**). The mean SctO_2_ per mmHg CVP is shown after stratification of the data points according to the simultaneously obtained cardiac output (2.5–5 L/min, 5–7.5 L/min, >7.5 L/min) (**b**)

Subsequently, all CVP-SctO_2_ pairs were stratified according to the simultaneously obtained MAP (60–70 mmHg, 70–80 mmHg, 80–90 mmHg, 90–100 mmHg) (Fig. 2a). Multivariate linear regression revealed that CVP was a more important determinant of SctO_2_ than MAP, without important interaction between MAP and CVP (SctO_2_ = 0.01 × MAP − 0.20 × CVP + 0.001 × MAP × CVP + 65.55 %). Only when MAP was high (90–100 mmHg, 18 % of data points), was the negative influence of CVP on SctO_2_ less pronounced.

### Interaction between CVP and cardiac output as determinants of SctO_2_

Subsequently, all CVP-SctO_2_ pairs were stratified according to the simultaneously obtained cardiac output (2.5–5 L/min, 5–7.5 L/min, >7.5 L/min) (Fig. 2b). Multivariate linear regression revealed that CVP and cardiac output are independent determinants of SctO_2_, with some interaction between both (SctO_2_ = 1.86 × CO – 0.09 × CVP – 0.05 × CO × CVP + 60.04 %). When cardiac output was high (>7.5 L/min, 4 % of data points) there was no negative correlation between CVP and SctO_2_.

### Trend analysis

**Fig. 3:**
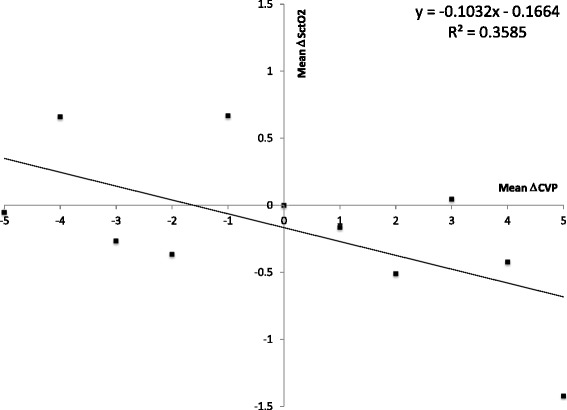
Trend analysis. The difference in central venous pressure (*ΔCVP*) and in cerebral tissue oxygen saturation (*ΔSctO*_*2*_) were calculated during 1-h time intervals during the 24-h study period. The mean ΔSctO_2_ is shown per mmHg mean ΔCVP

We calculated ΔCVP and ΔSctO_2_ during 1-h time intervals during the 24-h study period. We found significant correlation between mean ΔSctO_2_ and mean ΔCVP during these time intervals (ΔSctO_2_ = −0.11 × ΔCVP – 0.17, *R*^2^ 0.36) (Fig. 3).

### Outcome

At 180 days, 24 (50 %) of the study patients survived with CPC 1–2. Logistic regression revealed that a higher percentage of time with CVP >5 mmHg was associated with a lower chance of survival, with a good neurological outcome at 180 days (OR 0.96, 95 % CI 0.92–1.00, *p* = 0.04) (Table 1). In two separate backward multivariate logistic regression models, the negative association between the percentage of time with a CVP >5 mmHg and outcome persisted after correction for cardiac arrest variables (model 1) and hemodynamic variables (model 2) (Table 2).

**Table 1:** Odds ratios (OR) for survival with a good neurological outcome at 180 days (cerebral performance category 1–2) per percentage of time above each central venous pressure (CVP)

Percentage of time at different CVP values	OR	95 % CI	*P*
CVP >1 mmHg	0.19	0.02–2.30	0.19
CVP >2 mmHg	0.46	0.17–1.23	0.12
CVP >3 mmHg	0.69	0.47–1.01	0.05
CVP >4 mmHg	0.90	0.80–1.02	0.10
CVP >5 mmHg	0.96	0.92–1.00	0.04
CVP >6 mmHg	0.97	0.95–1.00	0.07
CVP >7 mmHg	0.98	0.97–1.01	0.19
CVP >8 mmHg	0.99	0.97–1.01	0.34
CVP >9 mmHg	0.99	0.98–1.01	0.45
CVP >10 mmHg	0.99	0.97–1.01	0.52
CVP > 15 mmHg	0.99	0.97–1.02	0.68
CVP > 20 mmHg	0.97	0.87–1.07	0.48

**Table 2:** Two separate multivariate models constructed using backward multivariate logistic regression

Model	Step 1	Final step		
	OR (*p* value)	OR	95 % CI	*P*
Model 1: cardiac arrest variables				
Age	0.99 (0.84)			
Bystander CPR, yes/no	3.75 (0.27)			
Shockable rhythm, yes/no	8.93 (0.02)	11.36	1.8–71.5	0.01
Time to ROSC	0.98 (0.63)			
Percentage time CVP >5 mmHg	0.94 (0.03)	0.94	0.89–0.99	0.02
Model 2: hemodynamic variables				
Percentage time MAP <65 mmHg	0.96 (0.03)	0.96	0.93–0.99	0.04
Ejection fraction, %	0.99 (0.56)			
Percentage time CVP >5 mmHg	0.95 (0.03)	0.95	0.90–0.99	0.03

In the first model, arrest variables were included (age, bystander cardiopulmonary resuscitation (CPR) <10 minutes, shockable rhythm, time to return of spontaneous circulation (ROSC), and percentage time central venous pressure (CVP) >5 mmHg) and in the second model, hemodynamic variables were included (mean, mean arterial pressure (MAP)/24 h, ejection fraction, mean CO/24 h, percentage time CVP >5 mmHg). Odds ratios (OR) for survival with a good neurological outcome at 180 days (cerebral performance category 1–2) are shown

## Discussion

This study is the first to support the hypothesis that venous cerebral congestion due to elevated CVP impairs cerebral oxygenation and may be detrimental for patients after CA. First, we showed that there is strong S-shaped negative correlation between CVP and SctO_2_. Based on a previous study, we consider SctO_2_ between 67 and 69 % to be optimal for patients after CA [3]. In this way our physiological model indicates that CVP >5 mmHg results in SctO_2_ < 67 % with progressively declining SctO_2_ with CVP >20 mmHg.

Correlation between paired MAP-CVP measurements and SctO_2_ was no better when compared to correlation between CVP and SctO_2_ alone. CVP remained an independent determinant of SctO_2_ in multivariate models with MAP and cardiac output, which argues against the hypothesis that high CVP would be a marker of worse hemodynamics with lower cardiac output or MAP and for this reason, lower SctO_2_. Only in exceptional cases with very high cardiac output (>7.5 L/min) SctO_2_ was CVP independent. We previously showed cerebrovascular autoregulation to be preserved in the majority of patients after CA and in these patients SctO_2_ is relatively MAP-independent [4]. In contrast to MAP, CVP is the passive result of fluid status, RV performance and intra-thoracic pressure and is less prone to active autoregulation. This probably explains why CVP was a more important determinant of SctO_2_ than MAP in the present study. Finally, trend analysis showed moderate correlation between mean ΔSctO_2_ and mean ΔCVP during the same 1-h time intervals. We hypothesize that this correlation is only moderate, because the majority of CVP changes during 1-h time intervals are small, and CVP changes in the plateau range of the S-shaped curve will not induce alterations of cerebral perfusion.

Although not completely comparable to our post-CA model, the concept of venous congestion being transmitted to jugular veins leading to cerebral ischemia is supported by reports that in patients on cardiopulmonary bypass, obstruction of the venous cannula elevates CVP followed by cerebral ischemia as detected with NIRS and cerebral dysfunction as detected with electroencephalogram (EEG) or bispectral index (BIS) monitors [7–13]. In experimental studies in pigs on bicaval cardiopulmonary bypass, 75 % occlusion of the superior vena cava flow increased CVP to 25 mmHg and ICP to 20 mmHg. This resulted in impaired cerebral perfusion as detected by lower sagittal sinus and cerebral tissue oxygen saturation. Both vasopressors and partial relief of the obstruction restored cerebral perfusion pressure and SctO_2_ to baseline values. However, only partial relief of the obstruction reduced ICP [8]. In agreement with our findings, the influence of CVP on cerebral perfusion was most pronounced in low arterial flow states. In contrast, 50 % obstruction of the flow increased CVP to 20 mmHg without significant decline of the cerebral perfusion, which is in line with our results showing that mainly CVP above a critical threshold of 20 mmHg result in pronounced cerebral hypoperfusion [9].

Additionally, in patients with traumatic brain injury, the application of positive end-expiratory pressure (PEEP) resulted in elevations of CVP, ICP and reductions of cerebral perfusion pressure, blood flow in the middle cerebral artery and cerebral desaturation [14–17]. The detrimental effects of PEEP were more pronounced in patients with normal respiratory system compliance, lower CVP, reduced intracranial compliance and low ICP, all of which favor the transduction of intrapulmonary to intravascular and intracranial pressure.

Second, we showed that the chance of survival with a good neurological outcome decreases per 1 % of extra time patients have CVP >5 mmHg. In line with our results, it has been shown that right ventricular dysfunction predicts poor outcome in patients after CA independent of left ventricular function (21). We acknowledge that many confounders may bias the link between CVP and outcome. It can be assumed that patients with higher CVP may have more preexisting chronic pulmonary disease, more frequent acute respiratory distress syndrome (ARDS)/acute *cor pulmonale*, lower arterial oxygen content, larger myocardial infarctions or more systolic left ventricular dysfunction, all of which by themselves may negatively affect the outcome or influence decisions on withdrawal of ICU therapy. Disproving this theory, the negative association between CVP and outcome persisted in a multivariate model after correction for hemodynamic variables, including ejection fraction and MAP. Moreover, our physiological model supports the hypothesis that cerebral hypoxia at least partially explains the link between venous cerebral congestion and worse outcome. Finally, the arterial PaO_2_ was not lower in patients with an average CVP above the median of 10 mmHg.

Potentially, these results have important consequences for routine clinical practice. The attention to CVP in modern critical care is waning because it is now generally accepted that it is a poor and static predictor of fluid responsiveness in mechanically ventilated patients [18]. Although we acknowledge that it will not be feasible to maintain normal CVP (<5 mmHg) in the majority of the mechanically ventilated post-CA patients, our results indicate that it is at least worth the effort to avoid fluid overload, treat right ventricular dysfunction appropriately and manage acute *cor pulmonale* by a lung-conserving ventilation strategy in order to keep CVP below 20 mmHg. In line with our results, a recent study showed an association between RV dysfunction and neurological outcome after cardiac arrest [19]. Future goal-directed treatment protocols for patients after CA should not only target a specific MAP but also focus on the other determinants of cerebral perfusion, including SVO_2_ and CVP.

We acknowledge that our study has some limitations including the lack of direct ICP measurements, invasive bulb oximetry (which was replaced by NIRS) and absence of carotid Doppler measurements to directly measure cerebral pressure, saturation and perfusion. In particular, NIRS measurements assume a 30/70 partition between the arterial and venous blood compartment. It cannot be excluded that the venous compartment enlarges in patients with elevated CVP without any change in cerebral blood flow.

Second, this is a small pilot study and these results should be regarded as hypothesis generating. We cannot exclude the possibility that our results were inflated as the 1,949,108 data points were collected in 48 patients and therefore may not be considered independent of each other. The small sample size most likely explains why parameters such as bystander CPR and time to ROSC were not independently associated with outcome in our multivariate model. Moreover, the general concept of cerebral congestion should be validated in other populations including patients with traumatic brain injury, severe acute decompensated heart failure (ADHF) and patients with high intra-thoracic pressures. In patients with ADHF, elevated CVP, rather than impaired cardiac output or low MAP, was the main determinant of worsening renal function during decongestive treatment and it would be interesting to see whether elevated CVP also impairs cerebral perfusion in patients with ADHF [20].

Third, this was not a randomized controlled interventional trial aiming for a specific CVP. Therefore, it cannot be excluded that the link between CVP and outcome is due to confounders and it remains open to further investigation whether aiming for the lowest feasible CVP would improve cerebral perfusion and the outcome for these patients. Finally, CVP measurements were automatically and continuously recorded with 2-sec time intervals. This allowed us to analyze 1,949,108 paired CVP-SctO_2_ measurements but had the disadvantage that automatic recordings are poorly validated against manual measurements and that automatic measurements are not performed end-expiratory. However, we do not believe this had a major influence on our results, as a delay can also be expected between CVP and SctO_2_ changes.

## Conclusion

In conclusion, we showed that CVP above 20 mmHg results in lower brain saturation and is associated with worse outcome in patients under therapeutic hypothermia after CA. This pilot study provides strong evidence for the first time that venous cerebral congestion may be detrimental for patients after CA. Based on our results, CVP >20 mmHg should be avoided in patients after CA.

## Key messages

- CVP >20 mmHg results in lower brain saturation and is associated with worse outcome in patients under therapeutic hypothermia after CA
- CVP >20 mmHg should be avoided in patients after CA

## Additional file

Additional file 1: Baseline Characteristics. (DOCX 96 kb)

## Abbreviations

ADHF: acute decompensated heart failure
CA: cardiac arrest
CO: cardiac output
CPC: cerebral performance category
CPR: cardiopulmonary resuscitation
CVP: central venous pressure
ICP: intracranial pressure
MAP: mean arterial pressure
NIRS: near infrared spectroscopy
OR: odds ratio
PaO_2_: partial pressure of oxygen
PEEP: positive end-expiratory pressure
ROSC: return of spontaneous circulation
SctO_2_: cerebral tissue oxygen saturation
SVO_2_: mixed venous blood oxygen saturation
