# Supplementary material for: Is venous congestion associated with reduced cerebral oxygenation and worse neurological outcome after cardiac arrest?
Source: Crit Care. 2016 May 15;20:146. doi: 10.1186/s13054-016-1297-2 (PMC4868016; doi:10.1186/s13054-016-1297-2)
Supplement: Additional file 1: — Baseline Characteristics. (DOCX 96 kb) [file 13054_2016_1297_MOESM1_ESM.docx]

|  | **All** | **Mean Central Venous Pressure/24hour** | | |
| --- | --- | --- | --- | --- |
|  |  | **Below Median**  **(<10.1 g/dl)** | **Above**  **Median**  **(>10.1 g/dl)** | **p-value** |
|  |  |  |  |  |
| **Number patients** | 48 | 24 | 24 |  |
| **Demographics** | | | | |
| Age (years) | 62±13 | 63±15 | 62±12 | 0.76 |
| Male (%) | 59 | 52 | 67 | 0.31 |
| **Resuscitation Parameters** | | | | |
| Bystander CPR<10 min (%) | 84 | 90 | 78 | 0.28 |
| Duration ALS (min) | 16±14 | 14±14 | 18±14 | 0.35 |
| Initial Rhythm Shockable (%) | 68 | 70 | 67 | 0.84 |
| **Hemodynamic and respiratory Parameters** | | | | |
| Mean Heartrate/24 hour (BPM) | 68±15 | 68±17 | 68±13 | 0.91 |
| Echocardiographic LVEF (%) | 38±17 | 38±17 | 38±17 | 0.90 |
| Mean MAP/24 hour (mmHg) | 77±8 | 76±8 | 78±8 | 0.39 |
| Mean SVO2/24 hour (%) | 67±10 | 70±8 | 64±10 | 0.06 |
| Mean CO/24 hour (l/min) | 3.70±1.24 | 3.70±1.16 | 3.73±1.37 | 0.93 |
| Mean CVP/24 hour (mmHg) | 10±4 | 7±2 | 13±2 | 0.00 |
| Mean mPAP/24 hour (mmHg) | 26±5 | 23±4 | 28±5 | 0.02 |
| Mean PaO2/24 hour (mmHg) | 100±19 | 105±19 | 95±17 | 0.05 |
| Mean PaCO2/24 hour (mmHg) | 41±4 | 39±4 | 42±4 | 0.07 |
|  |  |  |  |  |
